# Abordagem de Bioinformática e Biologia de Sistemas para Identificar a Ligação Patogenética entre Insuficiência Cardíaca e Sarcopenia

**DOI:** 10.36660/abc.20220874

**Published:** 2023-10-16

**Authors:** Rui Xu, Ling-ling Ma, Shuai Cui, Ling Chen, Hong Xu

**Affiliations:** 1 Gerontology center People’s Hospital of Xinjiang Uygur Autonomous Region Urumqi China Gerontology center, People’s Hospital of Xinjiang Uygur Autonomous Region, Urumqi – China

**Keywords:** Sarcopenia, Insuficiência Cardíaca, Biologia Computacional, Genes

## Abstract

**Fundamento:**

Apesar das evidências crescentes de que pacientes com insuficiência cardíaca (IC) são suscetíveis à sarcopenia, o motivo da associação não é bem compreendido.

**Objetivo:**

O objetivo deste estudo é explorar ainda mais o mecanismo molecular de ocorrência desta complicação.

**Métodos:**

Conjuntos de dados de expressão gênica para HF (GSE57345) e Sarcopenia (GSE1428) foram obtidos do banco de dados Gene Expression Omnibus (GEO). Genes diferencialmente expressos (DEGs) foram identificados usando pacotes ‘edgeR’ e “limma” de R, e suas funções foram analisadas usando Gene Ontology (GO) e a Enciclopédia de Genes e Genomas de Kyoto (KEGG). Redes de interação proteína-proteína (PPI) foram construídas e visualizadas usando Search Tool for the Retrieval of Interacting Genes (STRING) e Cytoscape. Os genes hub foram selecionados usando o plugin cytoHubba e validados com GSE76701 para IC e GSE136344 para Sarcopenia. As vias relacionadas e os mecanismos moleculares dos genes hub foram realizados pela análise de enriquecimento de genes (GSEA). As análises estatísticas foram realizadas no software R. P < 0,05 foi considerado estatisticamente significativo.

**Resultados:**

Foram encontrados 114 DEGs comuns. As vias relacionadas ao fator de crescimento, secreção de insulina e cGMP-PKG estavam enriquecidas tanto na IC quanto na sarcopenia. Descobriu-se que CYP27A1, KCNJ8, PIK3R5, TIMP2, CXCL12, KIT e VCAM1 são genes hub significativos após validação com GSEA enfatizando a importância dos genes hub na regulação da resposta inflamatória.

**Conclusão:**

Nosso estudo revela que a IC e a Sarcopenia compartilham vias e mecanismos patogênicos comuns. Estes achados podem sugerir novas direções para pesquisas futuras sobre a patogênese subjacente.

## Figura Central

**Figure:**
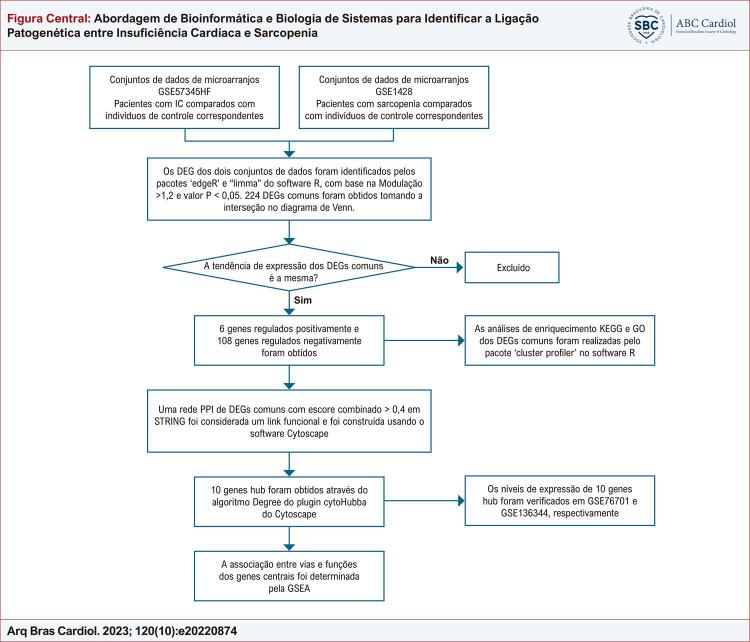
: Abordagem de Bioinformática e Biologia de Sistemas para Identificar a Ligação Patogenética entre Insuficiência Cardíaca e Sarcopenia

Fluxograma do projeto de pesquisa.

## Introdução

O envelhecimento global das populações em todo o mundo aumenta a prevalência de doenças relacionadas com a idade, como a IC, o que onera significativamente os sistemas de saúde.^1^ A etiologia da IC é complexa e multifatorial, resultando na redução da capacidade funcional, muitas vezes com mau prognóstico. A sarcopenia foi identificada como um potencial preditor extracardíaco de pior prognóstico em pacientes com IC.^2^

A sarcopenia é um distúrbio progressivo em que os indivíduos afetados experimentam a perda progressiva e debilitante de massa muscular, contribuindo, em última análise, para altas taxas de fragilidade entre as populações mais idosas.^3^ Está associada a um risco aumentado de quedas, osteoporose, perda de independência e aumento da mortalidade.^4^ A perda muscular é frequentemente descrita como um tipo de sarcopenia secundária, às vezes sob o termo “caquexia” em pacientes com IC.^5^ No entanto, embora esta perda de massa muscular esquelética associada à idade continue a ser uma grande preocupação para pacientes idosos com IC, os mecanismos subjacentes à co-ocorrência de sarcopenia e IC são pouco compreendidos.

Uma análise de genes e vias comuns pode fornecer informações sobre a coexistência de IC e Sarcopenia. Assim, analisamos genes hub comuns a ambos os distúrbios e previmos as vias associadas a esses genes por meio de análise bioinformática quantitativa de dados disponíveis publicamente. Os achados podem fornecer uma nova visão sobre os mecanismos subjacentes à co-ocorrência destes dois distúrbios comuns.

## Métodos

### Desenho do estudo e coleta de dados

Gene Expression Omnibus (GEO) é um repositório público de dados genômicos funcionais que suporta envios de dados compatíveis com MIAME. Ferramentas são fornecidas para ajudar os usuários a consultarem e baixar experimentos e perfis de expressão genética selecionados. Os conjuntos de dados de expressão gênica foram obtidos do banco de dados GEO usando os termos de pesquisa “Heart Failure” e “Sarcopenia”.^6,7^ Para inclusão, os critérios foram a presença de matrizes independentes com grandes tamanhos de amostra e dados humanos. Isso resultou na inclusão de dois conjuntos de dados, a saber, GSE573457 e GSE1428.^8^ O conjunto de dados GSE57345 incluiu dados de sequenciamento de RNA de 177 pacientes com IC e 136 controles saudáveis da Filadélfia, enquanto o conjunto de dados GSE1428 continha dados de sequenciamento de RNA de amostras do músculo vasto lateral de 12 pacientes com Sarcopenia (70-80 anos) e 10 jovens controles saudáveis (19-25 anos) de Boston.

### Identificação de genes expressos diferencialmente com software R

Os dados de GSE57345 e GSE1428 foram normalizados, e os DEGs entre amostras de pacientes e controle foram identificados com o pacote R ‘edgeR’ e ‘limma’.^9^ Modulações foram determinadas para a expressão dos genes individuais, com genes mostrando modulações > 1,2 e valor p < 0,05 classificado como DEGs. Genes comuns à Sarcopenia e IC foram obtidos pela sobreposição dos dois conjuntos de DEGs. O pacote R ‘Venn Diagram’ foi usado para obter seus DEGs comuns.^10^ Em seguida, sobrepusemos os genes relacionados de HF e Sarcopenia para obter genes comuns para análise posterior.

### Anotação funcional e análise de enriquecimento de via

Uma análise funcional adicional dos DEGs comuns foi conduzida pela avaliação de anotações GO e vias enriquecidos em KEGG usando o pacote ‘cluster’ em R.^11^ As anotações GO se enquadram em três categorias, a saber, processo biológico (BP), componente celular (CC) e função molecular (MF). Valor de P < 0,05 foi utilizado como limite de significância. Construção de rede PPI e identificação de genes hub

As redes PPI para os DEGs comuns foram então criadas em STRING com visualização pelo Cytoscape 3.9.0.^12^ Escores de confiança> 0,4 foram definidas para valores intermediários. O plugin Cytoscape, CytoHubba, foi utilizado para filtrar os genes hub na rede PPI usando o algoritmo de Degree.^13^

### Análise de enriquecimento de conjunto genético

O GSEA foi usada para determinar as associações entre vias e funções dos genes hub.^14^ Os níveis de significância foram estabelecidos em valores nominais de p <0,05, escores de enriquecimento normalizados (NES) > 1 e valores q de taxa de falsos positivos (FDR) <0,25.

### Validação da expressão de genes hub em outros conjuntos de dados

Os níveis de mRNA dos genes hub foram então verificados para GSE76701^15^ e GSE136344.^16^ GSE76701 continha 4 indivíduos com IC e 4 controles, enquanto GSE136344 continha 19 indivíduos com Sarcopenia e 11 controles. O teste T avaliou as diferenças entre os dois conjuntos de dados com um valor p <0,05 considerado significativo.

### Análise estatística

Este estudo conduziu todas as análises estatísticas usando o software R (versão 4.1.2; https://www.r-project.org/). A distribuição normal dos diferentes parâmetros foi verificada pelo teste de Kolmogorov-Smirnov. As diferenças entre os grupos foram avaliadas pelo teste t não pareado de Student. Um valor de p < 0,05 foi considerado significativo.

## Resultados

### Identificação de DEGs

**Figura 1:**
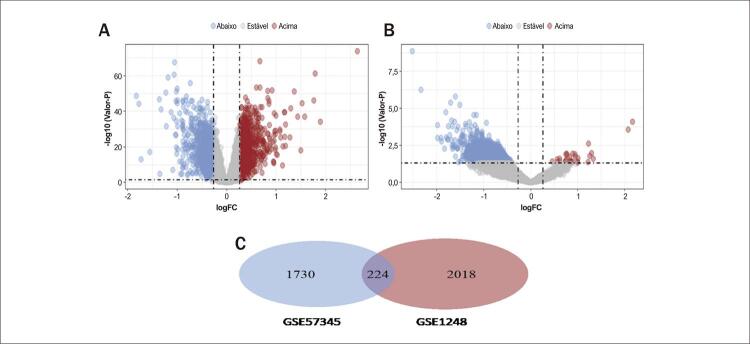
– Volcano plot e diagrama de Venn. A) Volcano plot de GSE57345. B) Volcano plot de GSE1428. Os genes regulados positivamente estão marcados em vermelho claro; genes regulados negativamente estão marcados em azul claro. C) Os dois conjuntos de dados mostraram uma sobreposição de 224 DEGs.

O fluxograma desta pesquisa foi apresentado na Figura Central. Todos os dados de dois conjuntos de dados independentes (GSE57345: HF e GSE1428: Sarcopenia) foram obtidos do GEO. Os dados do microarray foram normalizados e os DEGs foram identificados (1954 em GSE57345 e 2242 em GSE1428). Para melhor visualização, os DEGs para HF e Sarcopenia foram apresentados como volcano plots (Figuras 1A, B). 224 DEGs comuns a ambos os grupos foram identificados utilizando o diagrama de Venn (Figura 1C). Os genes que apresentaram diferentes tendências de expressão nos conjuntos de dados GSE57345 e GSE1428 foram descartados da análise, deixando 114 DEGs restantes.

### Análises de vias GO e KEGG

**Figura 2:**
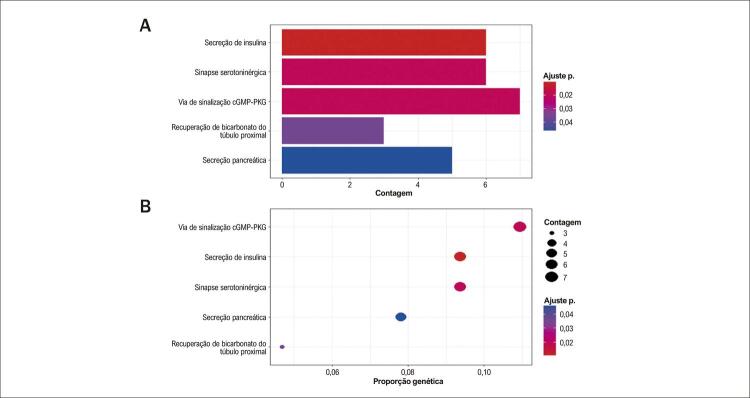
– A) Com base no valor adj P, o gráfico de barras mostra as principais vias KEGG entre sarcopenia e IC. B) As principais vias de enriquecimento do KEGG foram apresentadas como mapas conceituais.

**Figura 3:**
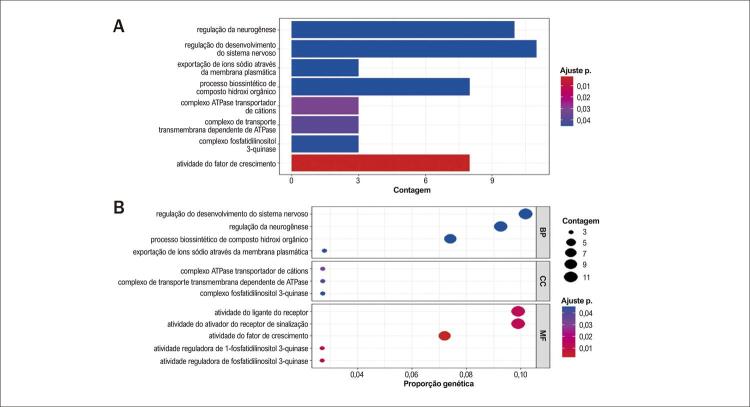
– A) Com base no valor de ajuste P, o gráfico de barras mostra as principais vias GO entre sarcopenia e IC em relação à função molecular, processo biológico e componente celular. B) Os principais vias de enriquecimento do banco de dados GO foram apresentados como mapas conceituais.

As funções desses DEGs comuns foram exploradas usando análises de enriquecimento GO e KEGG no pacote ‘cluster profiler’ no software R. A análise KEGG indicou enriquecimento dos DEGs em vias relacionadas ao fator de crescimento, secreção de insulina e cGMP-PKG (Figura 2A, 2B). As análises GO mostraram que os genes foram enriquecidos principalmente na via do fator de crescimento (Figura 3A, 3B).

### Construção de rede PPI de DEGs comuns e identificação de genes hub

**Figura 4:**
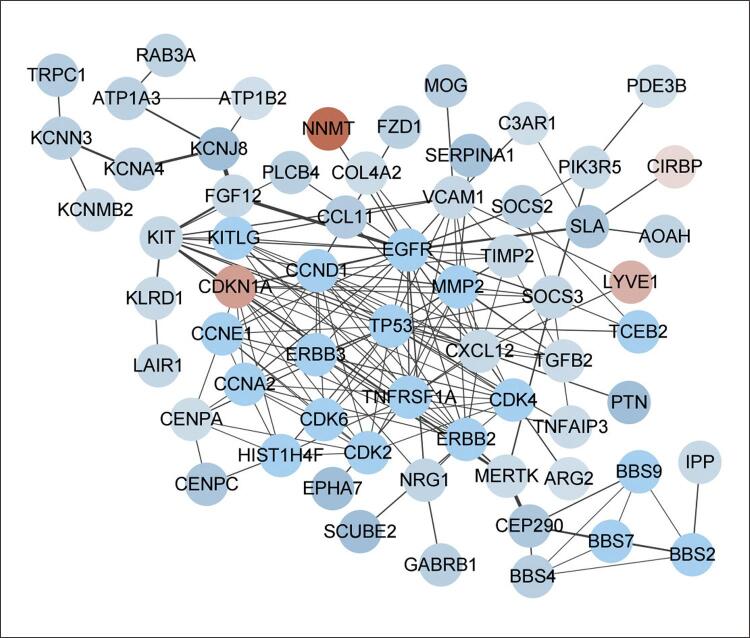
– Diagrama de rede PPI. Vermelho indica genes regulados positivamente e azul claro indica genes regulados negativamente.

**Figura 5:**
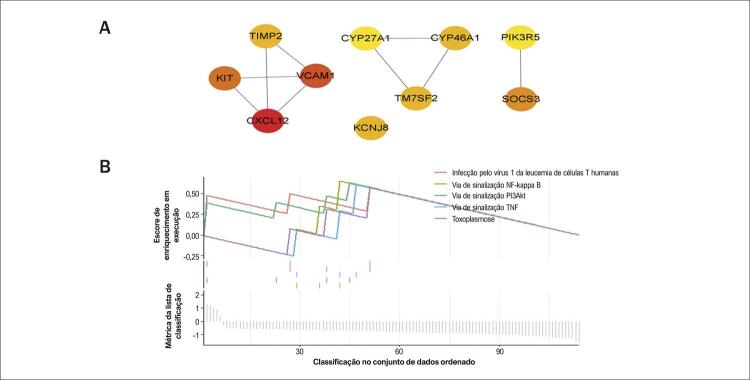
– A) Detecção de genes hub da rede PPIs de genes comuns. Os 10 genes hub destacados com base em seu grau. B) GSEA dos genes hub.

Os 114 DEGs comuns foram então importados para o STRING, com o arquivo STRING posteriormente importado para o Cytoscape para visualização. A Figura 4 mostra a rede PPI, na qual podem ser vistos 64 nós e 180 bordas. Os 10 principais genes hub foram encontrados usando o plugin CytoHubba e avaliados pelo grau de CYP27A1, KCNJ8, PIK3R5, TM7SF2, TIMP2, CXCL12, KIT, VCAM1, CYP46A1 e VCAM1 (Figura 5A).

### Resultados GSEA de genes hub

O GSEA foi então utilizado para examinar as possíveis funções dos genes hub, juntamente com a identificação de vias afetadas pela expressão diferencial dos genes, levando assim à identificação de vias associadas ao desenvolvimento de IC e sarcopenia. Os resultados mostraram que os genes hub foram significativamente associados à ativação das vias de sinalização NF-kappa B e de sinalização TNF (Figura 5B).

### Validação de Genes Hub

**Figura 6:**
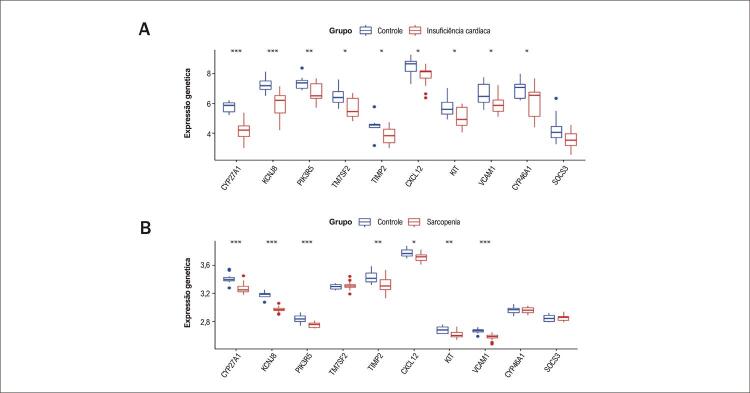
– Validação de genes hub. A) Os genes Hub foram validados em GSE76701 para IC. B) Os genes Hub foram validados em GSE136344 para Sarcopenia. *p < 0,05, **p < 0,01, ***p < 0,001.

Esses achados foram validados nos conjuntos de dados GEO GSE76701 para IC e GSE136344 para Sarcopenia. Em comparação com os controles, a interseção de 10 genes dos dois arquivos de matriz de conjuntos de dados revelou a regulação negativa significativa de 7 genes hub candidatos na IC (Figura 6A) e na Sarcopenia (Figura 6B). Esses genes hub foram CYP27A1, KCNJ8, PIK3R5, TIMP2, CXCL12, KIT e VCAM1.

## Discussão

Há evidências de que muitos pacientes com IC apresentam fadiga, deficiência nutricional, diminuição da capacidade de caminhar e redução da força muscular, conhecida como sarcopenia. A sarcopenia está associada ao envelhecimento e é caracterizada pela redução da resistência física e da massa muscular.^17^ A incidência de sarcopenia é maior em pacientes com IC em comparação com indivíduos controle da mesma idade, e esses pacientes frequentemente apresentam perda muscular mais rápida, o que compromete ainda mais sua função cardíaca.^2^ É, portanto, provável que a IC e a sarcopenia possam ter uma patogênese comum ou sobreposta. A elucidação desses mecanismos patogênicos é necessária para o desenvolvimento de tratamentos adequados.

Este estudo identificou 114 DEGs que se sobrepunham entre as duas doenças. As redes PPI e a subsequente validação destes DEGs sobrepostos identificaram 7 genes significativos, nomeadamente, CYP27A1, KCNJ8, PIK3R5, TIMP2, CXCL12, KIT e VCAM1. Como mostrado pelas análises de enriquecimento GO e KEGG, estes genes foram significativamente enriquecidos nas vias responsáveis pelo fator de crescimento, secreção de insulina e cGMP-PKG. As vias dos fatores de crescimento desempenham papéis importantes no desenvolvimento e manutenção da vasculatura, prevenindo o crescimento excessivo, a remodelação e a desestabilização por vários mecanismos de feedback.^18^ A via de secreção da insulina é fundamental para o metabolismo da glicose, e sua desregulação está associada ao diabetes, um conhecido fator de risco para IC e Sarcopenia.^19^ A via cGMP-PKG está envolvida na disfunção diastólica, associada à rigidez diastólica, relaxamento lento e redução da elasticidade dos cardiomiócitos.^20^

A GSEA indicou a associação de vias relacionadas à inflamação, incluindo as vias de sinalização NF-kappa B e TNF, com a patogênese da IC e da sarcopenia. Ambos os distúrbios estão associados à inflamação crônica, como observado nos níveis elevados de citocinas pró-inflamatórias, como TNF-a, IL-6 e IL-12. Estes aumentam a adiposidade visceral e reduzem a massa e a força muscular, aumentando o risco de IC.^21,22^ Nossos achados sugerem que os genes hub estão intimamente envolvidos com processos relacionados à inflamação mediados pelas vias de sinalização identificadas e contribuem para o desenvolvimento de IC e Sarcopenia. Considerando os genes hub, o CYP27A1 é um membro da família do citocromo P450 responsável por regular a homeostase do colesterol, convertendo o excesso de colesterol em ácido biliar.^23^ Ele também catalisa a 25-hidroxilação da vitamina D3, resultando na ativação funcional.^24^ Tanto a homeostase do colesterol quanto a vitamina D os níveis têm sido associados à patogênese e aos resultados da IC e da sarcopenia.^25,26^ O KCNJ8 é expresso pela maioria das células de mamíferos, onde regula os potenciais de membrana; níveis elevados são encontrados no coração, onde, juntamente com o SER2, forma um canal de potássio dependente de ATP. KCNJ8 tem sido associado a distúrbios cardiovasculares, incluindo vasomoção coronariana anormal e disfunção microvascular, doença cardíaca isquêmica e diabetes tipo 2.^27-29^ PIK3R5 está envolvido em muitos processos celulares, incluindo crescimento, proliferação, diferenciação, motilidade, tráfego intracelular e sobrevivência. Também foi proposto como biomarcador para hipertensão e diabetes mellitus.^30,31^ Foi relatado que níveis elevados de pressão arterial e glicose estão associados ao aumento da incidência de IC e sarcopenia.^32,33^ TIMP2, juntamente com outros membros da família de genes TIMP, inibem metaloproteinases de matriz (MMPs).^34^ MMPs, incluindo MMP-1, -2, -3, -9 e -19, são peptidases que degradam a matriz extracelular. TIMP2 e essas MMPs podem controlar a homeostase da matriz, modulando, especialmente, a produção e degradação de colágeno, que é conhecido por desempenhar um papel importante na patogênese da IC.^35^ A interrupção do equilíbrio MMP/TIMP2 no envelhecimento dos músculos esqueléticos afeta adversamente a função metabólica de a matriz extracelular e a produção excessiva de colágeno; estes, por sua vez, influenciam a massa e a função muscular e podem levar à sarcopenia.^36^ CXCL12 é um ligante de um receptor acoplado à proteína G e é conhecido por estar envolvido em várias atividades celulares, incluindo respostas imunológicas e inflamatórias, embriogênese, homeostase de tecido e carcinogênese e metástase.^37^ O CXCL12 é relatado como um elo importante entre inflamação e fibrose e foi proposto como um alvo para o tratamento da IC.^38^ Na sarcopenia, o CXCL12 influencia o desenvolvimento e funcionamento de osteoblastos, osteoclastos, células satélites e mioblastos, todos necessários para manter a homeostase muscular.^39^ O KIT codifica um receptor tirosina quinase que regula a proliferação e sobrevivência celular, bem como o desenvolvimento de mastócitos, gametogênese e melanogênese.^40^ O KIT é supostamente fortemente expresso no tecido cardíaco e parece ser envolvido na IC.^41^ O KIT promove a fosforilação de MAPK1/ERK2 durante a mitofagia.^42^ As interrupções na mitofagia, a degradação autofágica de mitocôndrias disfuncionais, estão associadas à atrofia das fibras musculares na sarcopenia.^43^ VCAM1 pertence à superfamília das imunoglobulinas e codifica uma sialoglicoproteína expressa em superfícies endoteliais após ativação de citocinas. Está envolvido na resposta imune e na promoção do direcionamento das células imunes para locais de inflamação.^44^ As vias imunológicas e inflamatórias estão associadas à patogênese da sarcopenia e da IC.^21,22^ Assim, os genes hub identificados e suas vias de sinalização associadas provavelmente serão intimamente envolvido na patogênese da IC e da sarcopenia.

No entanto, este estudo tem várias limitações. O estudo retrospectivo concentrou-se em um conjunto de dados de expressão gênica com um tamanho de amostra relativamente pequeno, levando potencialmente a um viés de seleção. Também é possível que genes significativos tenham sido negligenciados durante as diferentes etapas do processo de seleção. Investigações futuras devem utilizar amostras maiores e avaliar modelos celulares e animais para verificação.

## Conclusões

Foram identificados DEGs comuns associados à IC e à sarcopenia, e suas funções e interações foram analisadas por redes de enriquecimento e PPI. Os resultados indicaram que ambas as doenças tinham muitas vias patogénicas comuns, possivelmente sob o controle dos genes hub identificados, ilustraram o possível mecanismo da sarcopenia secundária à IC e identificaram novos genes candidatos que poderiam ser utilizados como biomarcadores ou como potenciais alvos terapêuticos.
