# Supplementary material for: Abordagem de Bioinformática e Biologia de Sistemas para Identificar a Ligação Patogenética entre Insuficiência Cardíaca e Sarcopenia
Source: Arq Bras Cardiol. 2023 Oct 16;120(10):e20220874. [Article in Portuguese] doi: 10.36660/abc.20220874 (PMC10586817; doi:10.36660/abc.20220874)
Supplement: Translation

# Translation: Bioinformatics and Systems Biology Approach to Identify the Pathogenetic Link between Heart Failure and Sarcopenia

**Authors:** Rui Xu, Ling-ling Ma, Shuai Cui, Ling Chen, Hong Xu

**Affiliations:** 1 China Gerontology center, People’s Hospital of Xinjiang Uygur Autonomous Region, Urumqi – China

**Keywords:** Sarcopenia, Heart Failure, Computational Biology, Genes

## Abstract

**Background:**

Despite increasing evidence that patients with heart failure (HF) are susceptible to sarcopenia, the reason for the association is not well understood.

**Objective:**

The purpose of this study is to explore further the molecular mechanism of the occurrence of this complication.

**Methods:**

Gene expression datasets for HF (GSE57345) and Sarcopenia (GSE1428) were obtained from the Gene Expression Omnibus (GEO) database. Differentially expressed genes (DEGs) were identified using ‘edgeR’ and “limma” packages of R, and their functions were analyzed using Gene Ontology (GO) and the Kyoto Encyclopedia of Genes and Genomes (KEGG). Protein-protein interaction (PPI) networks were constructed and visualized using Search Tool for the Retrieval of Interacting Genes (STRING) and Cytoscape. Hub genes were selected using the plugin cytoHubba and validation with GSE76701 for HF and GSE136344 for Sarcopenia. The related pathways and molecular mechanisms of the hub genes were performed by Gene set enrichment analysis (GSEA). The statistical analyses were performed using R software. P < 0.05 was considered statistically significant.

**Results:**

A total of 114 common DEGs were found. Pathways related to growth factor, Insulin secretion and cGMP-PKG were enriched in both HF and Sarcopenia. CYP27A1, KCNJ8, PIK3R5, TIMP2, CXCL12, KIT, and VCAM1 were found to be significant hub genes after validation, with GSEA emphasizing the importance of the hub genes in the regulation of the inflammatory response.

**Conclusion:**

Our study reveals that HF and Sarcopenia share common pathways and pathogenic mechanisms. These findings may suggest new directions for future research into the underlying pathogenesis.

## Introduction

The overall aging of populations worldwide is leading to an increased prevalence of age-related disorders such as HF, which burdens healthcare systems significantly.^1^ The etiology of HF is complex and multifactorial, resulting in reduced functional capacity, often with poor prognosis. Sarcopenia has been identified as a potential extracardiac predictor of a poorer prognosis in HF patients.^2^

Sarcopenia is a progressive disorder wherein affected individuals experience the progressive, debilitating loss of muscle mass, ultimately contributing to high rates of frailty among older populations.^3^ It is associated with an increased risk of falling, osteoporosis, loss of independence, and increased mortality.^4^ Muscle wasting is frequently described as a type of secondary sarcopenia, sometimes under the term “cachexia” in patients with HF.^5^ Nevertheless, while this age-associated loss of skeletal muscle mass remains a major concern for elderly patients with HF, the mechanisms underlying the co-occurrence of sarcopenia and HF are poorly understood.

An analysis of common genes and pathways may provide insight into the coexistence of HF and Sarcopenia. Thus, we analyzed hub genes common to both disorders and predicted the pathways associated with these genes by quantitative bioinformatic analysis of publicly available data. The findings may provide fresh insight into the mechanisms underlying the co-occurrence of these two common disorders.

## Methods

### Study design and data collection

Gene Expression Omnibus (GEO) is a public functional genomics data repository supporting MIAME-compliant data submissions. Tools are provided to help users query and download experiments and curated gene expression profiles. Gene expression datasets were obtained from the GEO database using the search terms “Heart Failure” and “Sarcopenia.”^6^ For inclusion, the criteria were the presence of independent arrays with large sample sizes and human data. This resulted in the inclusion of two datasets, namely, GSE57345^7^ and GSE1428.^8^ The GSE57345 dataset included RNA-sequencing data from 177 patients with HF and 136 healthy controls from Philadelphia, while the GSE1428 dataset contained RNA-sequencing data of vastus lateralis muscle samples from 12 patients with Sarcopenia (70-80 years old) and 10 young healthy controls (19-25 years old) from Boston.

### Identification of differentially expressed genes with R software

The data from GSE57345 and GSE1428 were normalized, and DEGs between patient and control samples were identified with the R package ‘edgeR’ and ‘limma’..^9^ Fold changes were determined for the expression of the individual genes, with genes showing Fold changes > 1.2 and P-value < 0.05 classified as DEGs. Genes common to Sarcopenia and HF were obtained by overlapping the two sets of DEGs. R package ‘VennDiagram’ was used to obtain their common DEGs.^10^ We then overlapped the related genes of HF and Sarcopenia to obtain common genes for further analysis.

### Functional annotation and pathway enrichment analysis

Further functional analysis of the common DEGs was conducted by the assessment of GO annotations and KEGG enriched pathways using the ‘cluster’ package in R.^11^ GO annotations fall into three categories, namely, biological process (BP), cellular component (CC) and molecular function (MF). P value < 0.05 was used as the threshold of significance.

### PPI network construction and identification of hub genes

The PPI networks for the common DEGs were then created in STRING with visualization by Cytoscape 3.9.0.^12^ Confidence scores > 0.4 were set to intermediate values. The Cytoscape plugin, CytoHubba, was used for filtering the hub genes in the PPI network using the algorithm of Degree.^13^

### Gene set enrichment analysis

GSEA was used to determine the associations between pathways and functions of the hub genes.^14^ Significance levels were set at nominal p values of < 0.05, normalized enrichment scores (NES) > 1, and false positive rate (FDR) q values of < 0.25.

### Validation of hub genes expression in other data sets

The mRNA levels of the hub genes were then verified for GSE76701^15^ and GSE136344.^16^ GSE76701 contained 4 HF subjects and 4 controls, while GSE136344 contained 19 Sarcopenia subjects and 11 controls. T-test assessed differences between the two data sets with a p-value < 0.05 considered significant.

### Statistical analysis

This study conducted all statistical analyses using R software (version 4.1.2; https://www.r-project.org/). The normal distribution of different parameters was verified with the Kolmogorov-Smirnov test. Differences between the groups were evaluated using Student’s unpaired t-test. A value of p < 0.05 was considered significant.

## Results

### Identification of DEGs

The research flowchart of this research was shown in the Central Figure. All data from two independent datasets (GSE57345: HF and GSE1428: Sarcopenia) were obtained from the GEO. The microarray data were normalized, and the DEGs were identified (1954 in GSE57345 and 2242 in GSE1428). For better visualization, the DEGs for HF and Sarcopenia were presented as volcano plots (Figures 1A, B). 224 DEGs common to both groups were identified using the Venn diagram (Figure 1C). Genes that showed different trends in expression in the GSE57345 and GSE1428 datasets were discarded from the analysis, leaving 114 DEGs remaining.

**Figure 1:** – Volcano diagram and Venn diagram. A) Volcano map of GSE57345. B) Volcano map of GSE1428. Upregulated genes are marked in light red; downregulated genes are marked in light blue. C) The two datasets showed an overlap of 224 DEGs.

### GO and KEGG Pathway Analyses

The functions of these common DEGs were explored using GO and KEGG enrichment analyses in the ‘cluster profiler’ package in R software. The KEGG analysis indicated enrichment of the DEGs in pathways related to growth factor, Insulin secretion, and cGMP-PKG (Figure 2A, 2B). GO analyses showed that the genes were mainly enriched in the growth factor pathway (Figure 3A, 3B).

**Figure 2:** – A) Based on the adj p value, the bar plot shows the Top KEGG pathways between sarcopenia and HF. B) The top enrichment pathways from KEGG were presented as bubble maps.

**Figure 3:** – A) Based on the adj P value, The bar plot shows the top GO pathways between sarcopenia and HF regarding molecular function, biological process, and cellular component. B) The top enrichment pathways from GO database were presented as bubble maps.

### PPI Network Construction of Common DEGs and Identification of Hub Genes

The 114 common DEGs were then imported into STRING, with the STRING file subsequently imported into Cytoscape for visualization. Figure 4 shows the PPI network, in which 64 nodes and 180 edges can be seen. The top 10 hub genes were found using the CytoHubba plugin and assessed by the degree to be CYP27A1, KCNJ8, PIK3R5, TM7SF2, TIMP2, CXCL12, KIT, VCAM1, CYP46A1, and VCAM1 (Figure 5A).

**Figure 4:** – PPI network diagram. Red indicates up-regulated genes and light blue indicates down-regulated genes.

**Figure 5:** – A) Detection of hub genes from the PPIs network of common genes. The highlighted 10 hub genes based on their degree. B) GSEA of the hub genes.

### GSEA Results of Hub Genes

GSEA was then used to examine the possible functions of the hub genes, together with identifying pathways affected by the differential expression of the genes, thus leading to the identification of pathways associated with the development of HF and Sarcopenia. Results showed that the hub genes were significantly associated with activating the NF-kappa B signaling and TNF-signaling pathways (Figure 5B).

### Validation of Hub Genes

These findings were validated in the GEO datasets GSE76701 for HF and GSE136344 for Sarcopenia. Compared with controls, the intersection of 10 genes from the two matrix files of datasets revealed the significant downregulation of 7 candidate hub genes in HF (Figure 6A) and Sarcopenia (Figure 6B). These hub genes were CYP27A1, KCNJ8, PIK3R5, TIMP2, CXCL12, KIT, and VCAM1.

**Figure 6:** – Validation of hub genes. A) Hub genes were validated in GSE76701 for HF. B) Hub genes were validated in GSE136344 for Sarcopenia. *p < 0.05, **p < 0.01, ***p < 0.001.

## Discussion

There is evidence that many patients with HF experience fatigue, nutritional deficiency, decreased ability to walk, and reduced muscle strength, known as Sarcopenia. Sarcopenia is associated with aging and is characterized by reduced physical stamina and muscle mass.^17^ The incidence of Sarcopenia is higher in HF patients compared with age-matched control subjects, and these patients often show more rapid muscle loss, which further compromises their cardiac function.^2^ It is thus likely that HF and Sarcopenia may have a common or overlapping pathogenesis. The elucidation of these pathogenic mechanisms is necessary to develop suitable treatments.

This study identified 114 DEGs that overlapped between the two diseases. PPI networks and subsequent validation of these overlapping DEGs identified 7 significant genes, namely, CYP27A1, KCNJ8, PIK3R5, TIMP2, CXCL12, KIT, and VCAM1. As shown by GO and KEGG enrichment analyses, these genes were significantly enriched in pathways responsible for growth factor, Insulin secretion, and cGMP-PKG. Growth factor pathways play major roles in developing and maintaining the vasculature, preventing excess growth, remodeling, and destabilization by various feedback mechanisms.^18^ The insulin secretion pathway is key to glucose metabolism, and its dysregulation is associated with diabetes, a known risk factor for both HF and Sarcopenia.^19^ The cGMP-PKG pathway is involved in diastolic dysfunction, associated with diastolic stiffness, slow relaxation, and reduced elasticity of the cardiomyocytes.^20^

GSEA indicated the association of inflammation-related pathways, including the NF-kappa B and TNF-signaling pathways, with HF and Sarcopenia pathogenesis. Both disorders are associated with chronic inflammation, as seen in the raised levels of pro-inflammatory cytokines, such as TNF-a, IL-6, and IL-12. These enhance visceral adiposity and reduce muscle mass and strength, increasing the risk of HF.^21,22^ Our findings suggest that the hub genes are closely involved with inflammation-related processes mediated by the identified signaling pathways and contribute to the development of HF and Sarcopenia.

Considering the hub genes, CYP27A1 is a member of the cytochrome P450 family responsible for regulating cholesterol homeostasis by converting excess cholesterol to bile acid.^23^ It also catalyzes 25-hydroxylation of vitamin D3, resulting in functional activation.^24^ Both cholesterol homeostasis and vitamin D levels have been linked to the pathogenesis and outcomes of HF and Sarcopenia.^25,26^ KCNJ8 is expressed by most mammalian cells, where it regulates membrane potentials; high levels are found in the heart, where it, together with SER2, forms an ATP-dependent potassium channel. KCNJ8 has been linked with cardiovascular disorders, including abnormal coronary vasomotion and microvascular dysfunction, ischemic heart disease, and type 2 diabetes.^27-29^ PIK3R5 is involved in many cellular processes, including growth, proliferation, differentiation, motility, intracellular trafficking, and survival. It has also been proposed as a biomarker for hypertension and diabetes mellitus.^30,31^ Raised blood pressure and glucose levels are reported to be associated with increased incidence of HF and Sarcopenia.^32,33^ TIMP2, together with other members of the TIMP gene family, inhibit matrix metalloproteinases (MMPs).^34^ MMPs, including MMP-1, -2, -3, -9, and -19, are peptidases that degrade the extracellular matrix. TIMP2 and these MMPs can control homeostasis of the matrix, modulating, especially, collagen production and degradation, which is known to play an important role in HF pathogenesis.^35^ Disruption of the MMP/TIMP2 balance in aging skeletal muscles adversely affects the metabolic function of the extracellular matrix and excess collagen production; these, in turn, influence both muscle mass and function and can lead to Sarcopenia.^36^ CXCL12 is a ligand of a G-protein-coupled receptor and is known to be involved in various cellular activities, including immune and inflammatory responses, embryogenesis, tissue homeostasis, and carcinogenesis and metastasis.^37^ CXCL12 is reported to be an important link between inflammation and fibrosis and has been proposed as a target for the treatment of HF.^38^ In sarcopenia, CXCL12 influences the development and functioning of osteoblasts, osteoclasts, satellite cells, and myoblasts, all necessary for maintaining muscle homeostasis.^39^ KIT encodes a receptor tyrosine kinase that regulates cellular proliferation and survival, as well as mast cell development, gametogenesis, and melanogenesis.^40^ KIT is reportedly strongly expressed in heart tissue and appears to be involved in HF.^41^ KIT promotes the phosphorylation of MAPK1/ERK2 during mitophagy.^42^ Disruptions in mitophagy, the autophagic degradation of dysfunctional mitochondria, are associated with muscle fiber atrophy in sarcopenia.^43^ VCAM1 belongs to the immunoglobulin superfamily and encodes a sialoglycoprotein expressed on endothelial surfaces following cytokine activation. It is involved in the immune response and promoting immune cell targeting to inflammation sites.^44^ Immune and inflammatory pathways are associated with the pathogenesis of both sarcopenia and HF.^21,22^ Thus, the identified hub genes and their associated signaling pathways are likely to be closely involved in the pathogenesis of both HF and Sarcopenia.

However, this study has several limitations. The retrospective study focused on a gene expression dataset with a relatively small sample size, potentially leading to selection bias. It is also possible that significant genes might have been overlooked during the different steps of the selection process. Future investigations should use larger samples and assess both cellular and animal models for verification.

## Conclusions

To summarize, common DEGs associated with HF and sarcopenia were identified, and their functions and interactions were analyzed by enrichment and PPI networks. The findings indicated that both diseases had many common pathogenic pathways, possibly under the control of the identified hub genes, illustrated the possible mechanism of sarcopenia secondary to HF, and identified novel gene candidates who could be used as biomarkers or as potential therapeutic targets.
